# Roles of Dietary Phytoestrogens on the Regulation of Epithelial-Mesenchymal Transition in Diverse Cancer Metastasis

**DOI:** 10.3390/toxins8060162

**Published:** 2016-05-24

**Authors:** Geum-A. Lee, Kyung-A. Hwang, Kyung-Chul Choi

**Affiliations:** Laboratory of Biochemistry and Immunology, College of Veterinary Medicine, Chungbuk National University, Cheongju, Chungbuk 361-763, Korea; mmanuraa@gmail.com

**Keywords:** dietary phytoestrogens, DIM, kaempferol, resveratrol, genistein, epithelial-mesenchymal transition, cancer metastasis

## Abstract

Epithelial-mesenchymal transition (EMT) plays a key role in tumor progression. The cells undergoing EMT upregulate the expression of cell motility-related proteins and show enhanced migration and invasion. The hallmarks of EMT in cancer cells include changed cell morphology and increased metastatic capabilities in cell migration and invasion. Therefore, prevention of EMT is an important tool for the inhibition of tumor metastasis. A novel preventive therapy is needed, such as treatment of natural dietary substances that are nontoxic to normal human cells, but effective in inhibiting cancer cells. Phytoestrogens, such as genistein, resveratrol, kaempferol and 3,3′-diindolylmethane (DIM), can be raised as possible candidates. They are plant-derived dietary estrogens, which are found in tea, vegetables and fruits, and are known to have various biological efficacies, including chemopreventive activity against cancers. Specifically, these phytoestrogens may induce not only anti-proliferation, apoptosis and cell cycle arrest, but also anti-metastasis by inhibiting the EMT process in various cancer cells. There have been several signaling pathways found to be associated with the induction of the EMT process in cancer cells. Phytoestrogens were demonstrated to have chemopreventive effects on cancer metastasis by inhibiting EMT-associated pathways, such as Notch-1 and TGF-beta signaling. As a result, phytoestrogens can inhibit or reverse the EMT process by upregulating the expression of epithelial phenotypes, including *E*-cadherin, and downregulating the expression of mesenchymal phenotypes, including *N*-cadherin, Snail, Slug, and vimentin. In this review, we focused on the important roles of phytoestrogens in inhibiting EMT in many types of cancer and suggested phytoestrogens as prominent alternative compounds to chemotherapy.

## 1. Introduction

Phytochemicals are chemical compounds that occur naturally in plants, amounting to as many as 4000 different chemicals. Some phytochemicals have the biological significance of inhibiting the invasion of different species of plants by acting as toxic compounds [1,2]. This characteristic of phytochemicals has been utilized in curing diverse human diseases. A vast array of plant-derived natural compounds has been reported to have substantial chemopreventive effects against cancer, in opposition to the health risk of environmental carcinogens [3]. At present, inhibiting human carcinogenesis using plant-derived compounds is considered as a vital and urgent challenge, despite some phytochemicals having been used for targeting many forms of cancer as major sources of highly effective conventional drugs [4,5,6].

Among diverse groups of phytochemicals, phytoestrogens, which are plant-derived xenoestrogens and mostly found in soy, vegetables and fruits, are considered as strong sources of cancer-preventive phytonutrition to inhibit the development and progression of many types of cancer [4,7].

As a kind of xenoestrogen, phytoestrogens specifically have distinctive cancer-preventive effects on estrogen-related cancers. In general, sex hormones, including estrogens, have been known to be closely linked to the pathogenesis of several types of cancer in the reproductive organs [8,9,10]. Many diseases, like breast, ovarian, endometrial and cervical cancers, have been called estrogen-receptor (ER)-positive cancers, because the actions of estrogen related to cancer biology are mediated via ERs, which comprise ERα and ERβ, mostly present in the nucleus [11,12]. Independently of endogenous estrogens, selective estrogen receptor modulators (SERMs), which are a class of drugs that act on ER, act with agonistic or antagonistic actions in several target tissues [13]. Phytoestrogens, which are also a kind of SERM, are known to bind ER with affinities at least 10,000-times lower than that of 17β-estradiol (E2) and also act as ER agonists or antagonists [14]. The chemopreventive effects of phytoestrogens are associated with their antagonistic effects on ER [15]. In addition to the actions of phytoestrogens via ERs, they could have protective effects on the initiation and progression of estrogen-related cancers by specifically inhibiting the circulating precursors of estrogens [16,17,18,19,20]. In addition, they can inhibit crucial steroidogenic enzyme activity, including the conversion of E2 from circulating hormones, such as androgens and estrogen sulfate [20]. In estrogen biosynthesis and metabolism, phytoestrogens have been shown to inhibit several crucial enzymes in aromatase pathway, such as 17β- and 3β-hydroxysteroid dehydrogenase (HSD), which catalyze the dehydrogenation of 17-hydroxysteroids in steroidogenesis and control the interconversion of androstenedione and testosterone, and E2 and estrone, respectively [21,22].

Phytoestrogens are generally classified into four main classes: isoflavones (genistein, daidzein, kaempferol), lignans (secoisolariciresinol, matairesinol, pinoresinol, lariciresinol), coumestan (coumestrol) and stilbenes (resveratrol) [23,24]. Western populations have been known to intake more foods containing lignans, while Asian populations eat more soy foods containing isoflavones [25]. Lignans are included in diverse groups of non-flavonoid compounds widely distributed in whole grain cereals, beans, berries, nuts and various seeds [26,27]. A wealth of lignans exists as secoisolariciresinol, matairesinol, lariciresinol and pinoresinol, and they are converted into enterolignans by the intestinal microbiota to be absorbed into the human body [27,28]. Tea, fruits, vegetables and grains account for over 85% of the daily intake of lignans, such as matairesinol and secoisolariciresinol [29]. Isoflavones are naturally-occurring phenolic flavonoid compounds, known to act as phytoestrogens in mammals. Soybeans are the most common source of isoflavones among vegetables; the major isoflavones in soybean are genistein and daidzein, which are well-known phytoestrogens [30]. Coumestans occur mainly in bean sprouts during germination, and the main compound in this subgroup is coumestrol, mostly found in peas and beans [23]. Stilbenoids are hydroxylated derivatives of stilbene and belong to the family of phenylpropanoids [31]. Resveratrol is a typical stilbenoid, which is mainly found in grapes and wines [32].

**Figure 1:**
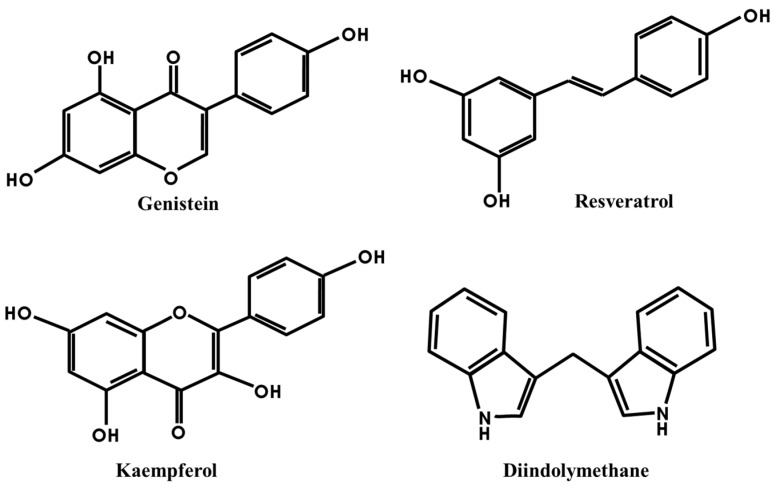
Chemical structures of phytoestrogens, genistein, resveratrol, kaempferol and 3,3′-diindolylmethane (DIM).

In the present article, we will review the effect of four kinds of phytoestrogens, genistein, resveratrol, kaempferol and 3,3′-diindolylmethane (DIM), on cancer progression via epithelial-mesenchymal transition (EMT). As shown in Figure 1, genistein, kaempferol and resveratrol are phenolic compounds: genistein and kaempferol are isoflavones, having a common flavone structure; resveratrol is a derivative of diphenylethane; and DIM is an active indole compound originated from indole-3-carbinol (I3C), an inactive form of indole. They are actively-studied phytoestrogens that have great potential to display anti-cancer effects.

EMT plays a key role in tumor progression. The cancer cells undergoing EMT increase the expression levels of cell motility-related proteins and show enhanced migration and invasion to other sites of the body, resulting in cancer metastasis [33]. It has been found that crucial EMT markers, such as *E*-cadherin and Snail, are identified to secure positive evidence of EMT, and several signaling pathways are associated with the induction of EMT process in cancer cells, including Not-1 and TGF-beta signalings. In the next section, the importance of EMT in cancer progression, diverse EMT markers and related signaling pathways are briefly introduced to further highlight the impact of these phytoestrogens in chemoprevention against cancer.

## 2. Epithelial-Mesenchymal Transition in Cancer Metastasis

According to the World Cancer Report 2014 of the World Health Organization (WHO), about 14.1 million new cases of cancer occurred globally in 2012, leading to 14.6% of all human deaths. Approximately 90% of all cancer-related deaths are reported to be associated with tumor metastasis [34]. The chance of having an invasive cancer in one’s lifetime is estimated to be 42% for men and 38% for women [35]. The characteristic of cancer malignancy and metastasis is the propagation of primary tumors through migrating to and invading the surrounding tissues [36]. Tumor cells have the potential to invade other tissues and to form metastasis through multiple steps known as malignant progression [37].

The program responsible for profound modification for metastasis that enables detaching from the junctions and dismissing the lateral cell-cell adhesions of cancer cells is EMT [38]. Tumor cells undergoing EMT display unique phenotypes, express higher levels of cell motility proteins and show promoted migration and invasion abilities [39].

EMT is associated with several major characteristics of cellular phenotypes. Through this process, epithelial cells change the morphology from a cobblestone-like monolayer with apical basal polarity to flat and spindle-shaped mesenchymal cells in the absence of polarization to gain the ability to move [40].

To acquire the moving ability, epithelial cells lose their ability to maintain the entire junction complex that connects them to the neighboring ones, of which basolateral surfaces are regularly spaced through membrane-associated specialized junctions [41]. In the process in which epithelial cells are switched to mesenchymal cells, the formation of a space where a barrier and rigidity are maintained is inhibited due to the lack of intercellular junctions [42]. A number of cells undergoing EMT develop interactions with the extracellular environment in localized areas of the carcinoma, where they involve the loss of intercellular cohesion, the disruption of extracellular matrix (ECM), modifications of the cytoskeleton, increased motility and invasion into the extracellular space [43]. Particularly, EMT is related to the expression of extracellular matrix proteases, such as matrix metalloproteases (MMPs) and urokinase-type plasminogen activator (uPA), which can degrade the ECM linked to the plasma membrane and localized to invadopodia during metastasis [44,45,46]. Meanwhile, EMT is a reversible process that can convert to its inverse process, called the mesenchymal-epithelial transition (MET). The cells undergoing MET increase cell-cell adhesion and return to epithelial phenotypes, which also play a role during embryonic development and pathological processes [47,48]. As a result, primary cancer cells lose cell-cell adhesion via EMT by *E*-cadherin repression, break through the basement membrane and enter the bloodstream through intravasation. Later, the circulating tumor cells exit the bloodstream to migrate to the specific metastatic sites where they undergo MET for clonal outgrowth [49].

A diversity of molecules associated with the process of EMT has been established, and some crucial molecules have been employed as biological markers to determine the process. As a typical molecule in the adherent junctions, *E*-cadherin, which is a transmembrane glycoprotein of the type I cadherin family and a crucial epithelial marker, has been found to inactivate and repress tumor progression by maintaining intact cell-cell interactions and inhibiting cell mobility, invasion and metastasis in human cancer [50,51]. The loss of *E*-cadherin expression is allowed for a critical step in the progression of invasive carcinoma by causing the EMT event. The loss of many epithelial markers (including *E*-cadherin, occludin, claudins and beta-catenin) induces the expression of mesenchymal markers (including *N*-cadherin, Snail, vimentin, R-cadherin and cadherin-11) and acquisition of mesenchymal characteristics, such as cell motility and invasion [52,53]. Additionally, the zinc-finger transcription factors, including Snail, Slug, and ZEB 1 and 2, have been shown to induce the EMT process by directly binding to the E-box of the *E*-cadherin promoter and suppressing the activity of *E*-cadherin [54], while Twist, another type of *E*-cadherin repressor, indirectly downregulates *E*-cadherin transcription [55].

It has been found that crucial EMT markers are associated with several signaling pathways in the induction of the EMT process in cancer cells. Snail has been found to be a critical factor in TGF-β signaling to resist cell death and to inhibit apoptosis [56]. As the most important factor that triggers EMT, TGF-β1 mediates the EMT process via numerous intracellular signaling pathways, including the Smad pathway, mitogen-activated protein kinases (MAPK), PI3K/Akt and small GTPases in HL-60 leukemia, Panc-1 human pancreatic and MDA-MB-231 breast cancer cells [57,58,59]. The overactivation of the TGF-β pathway in hepatocellular carcinoma cells confers a mesenchymal-like and an increased migratory capacity to the cells and finally contributes to tumor progression thorough the crosstalk with the chemokine CXCL12 pathway in liver tumor cells [60]. The Wnt signaling pathway activates β-catenin and several EMT-inducing transcription factors, such as Slug and Twist [34]. This signaling pathway induces cancer cell proliferation, motility and intravasation. Furthermore, in *in vivo* studies, the Wnt pathway displayed an important role in regulating EMT progression of colorectal cancer [61], and the Wnt-β-catenin was activated in the mesenchyme of the cardiac cushion during EMT in zebrafish and mouse embryo [62,63]. The cancer development of organs has been regulated by Notch-1 signaling, which directly promotes Snail, Slug and NF-κB in BxPC-3 human pancreatic cancer cell [64]. Notch-1 signaling also induced cell proliferation, survival and EMT by increasing NF-κB transcriptional activity in many human malignancies, including pancreatic and breast cancer cells [65,66]. In addition, the Hedgehog (Hh) signaling pathway is currently considered as a therapeutic target for anti-cancer treatment, because this pathway is abnormally activated in various types of cancer and contributes to tumor metastasis by inducing EMT. The misregulation of Hh signaling has been implicated as an important mediator in human pancreatic carcinoma, and specifically the sonic hedgehog pathway promotes metastasis and lymphangiogenesis via activation of Akt, EMT and the MMP-9 pathway in gastric cancers [67,68].

Recently, microRNAs (miRNAs) are being considered as an important regulator of EMT in various cancer cells. They incompletely bind to the 3′untranslated region (3′UTR) of mRNA to inhibit the translations [69]. The incomplete accordance between miRNAs and their targets allows the chances for miRNAs to control multiple genes. Moreover, miRNAs have been shown to play a crucial role during caner development and progression via the modulation of the expression of their target mRNA transcripts [70]. High miR-34a levels stimulate MET by reversing Snail and TCF-β-induced EMT [71]. As a negative regulator in the EMT process, miR-125a induced MET by the epidermal growth factor receptor (EGFR) signaling pathway [72]. miR-506 suppresses EMT, cell proliferation, migration and invasion by upregulating *E*-cadherin [73]. miR-138 also has a role in the inhibition of EMT and invasion in SKOV-3 ovarian cancer cells [74]. Another miRNA, miR-30a, was reported to suppress cell motility and EMT via targeting the expression of mesenchymal markers, thereby increasing the epithelial marker in A549 lung and BGC-823 gastric cancer cells [75,76]. On the other hand, miR-106a is associated with cell proliferation and tumor differentiation, and miR-7 is linked with metastasis and EMT [77]. In the case of miR-10b, its high expression upregulated the EMT and the expression of EMT-related proteins in metastatic tumors and induced the changed spindle-like morphology, cell migration and overexpression of *N*-cadherin, Snail, Slug and Twist [78,79]. Specifically, the miR-200 family is significant for reducing the ZEB levels, cell migration and TGF-β-induced EMT [80,81]. Expression of the miR-200 family was increased or decreased in the process of metastasis: miR-200 was downregulated in the EMT process, while it was upregulated during the re-epithelialization of distal metastasis [82].

## 3. Phytoestrogens and Their Actions on Cancer Cells Undergoing EMT

Since the dysregulation of proteins in signaling pathways involved in EMT is associated with cancer progression, they could be potentially targeted as prognostic markers or therapeutic targets of cancer metastasis [83]. Phytoestrogens having a chemopreventive effect on cancer progression seem to inhibit the EMT process through various channels.

### 3.1. Genistein

Genistein (40,5,7-trihydroxyisoflavone), having a heterocyclic diphenolic structure similar to estrogen, is a typical isoflavonoid found in a number of plants, including soybeans, peas, lentils and other beans [84,85]. As a phytoestrogen, it has an ability to bind and activate ERs, preferentially ERβ rather than ERα [86,87]. The higher binding affinity for ERβ of genistein has been associated with its action as an estrogen antagonist and having chemopreventive activity in estrogen-responsive cancers [88].

Anti-proliferative and chemopreventive effects of genistein have been extensively investigated in hormone-related, as well as non-hormone-related cancers in which genistein affects many crucial cellular functions related to carcinogenesis, including cell proliferation, apoptosis, cell cycle progression, migration, metastasis and invasion [89]. Recent studies have elucidated that genistein may have the potential to inhibit cancer metastasis by specifically regulating the EMT process via diverse signaling pathways.

Notch-1 signaling is an important pathway to upregulate the expression of EMT markers, ZEB1 and 2, Slug and vimentin, leading to the EMT, migration and drug resistance of pancreatic cancer cells [90,91]. In AsPC-1 pancreatic cancer cells, Notch-1 overexpression affected the expression of miRNAs: overexpression of Notch-1 led to increased expression of miR-21 and decreased expression of miR-200b. Re-expression of miR-200b led to inhibition of the EMT process by inducing decreased expression of ZEB1 and vimentin and increased expression of *E*-cadherin. Genistein treatment was found to attenuate the acquisition of EMT by AsPC-1 cells by promoting re-expression of miR-200b, which was repressed by Notch-1 signaling [90].

In a recent study, genistein suppressed the EMT of BG-1 ovarian cancer cells, which was activated by E2 and endocrine-disrupting chemicals, such as bisphenol A (BPA) and nonylphenol (NP), by downregulating the TGF-β pathway [92]. As a result, genistein not only suppressed the migration of BG-1 cells, but also diminished the expression of mesenchymal markers (vimentin) and metastasis markers (MMP-2 and cathepsin D) [92]. Genistein also effectively inhibited TGF-β-induced invasion and metastasis in the Panc-1 pancreatic cancer cell line through Smad4-dependent and independent pathways through p38 MAPK [58]. The EMT process was also reversed by genistein in the HepG2 hepatocellular carcinoma cell line by downregulating the nuclear factor of activated T cells 1 (NFAT1) [93]. NFAT1 was known to function in cell-autonomous actions, like invasion, migration, differentiation and proliferation in tumors [94]. Although the underlying mechanism has not been found yet, genistein suppressed invasive growth of LNCaP prostate cancer cells through the reversal of EMT, even at low concentrations (less than 15 micromoles/L genistein), which did not affect cell proliferation [95].

### 3.2. Resveratrol

Resveratrol (trans-3,4,5-trihydroxystilbene) is one of the stilbene phytoalexins, first found in the roots of the oriental medicinal plant *Polygonum cuspidatum* (Kojo-kon in Japanese) [96], and also exists in diverse vegetables, including berries, peanuts and red grape [97]. Resveratrol is known to be produced naturally when the plant is injured under attack by pathogens, such as bacteria or fungi [98]. Therefore, the proper infection of *Botrytis cinerea* (the fungus responsible for grey mold) is needed to obtain maximal concentrations of resveratrol within wine [99]. The characteristic of resveratrol as a phytoestrogen has been verified by its capability to mainly bind to ER and to regulate the transcription of estrogen-responsive target genes [100]. Many studies showed that resveratrol binds to ERβ with a higher affinity than to ERα, though it binds with 7000-fold lower affinity than E2, and that it acts as an agonist or an antagonist in the cells expressing ER [101,102]. Resveratrol can also regulate androgen receptor (AR)-mediated actions as a chemopreventive agent against prostate cancer: it inhibited androgen-stimulated cell growth and gene expression by repressing the expression and function of the AR in LNCaP prostate cancer cells [103].

In addition to its sex hormone-related actions, resveratrol has been found to be very helpful in inhibiting diabetes, heart disease and diverse cancers, because it possesses various bioactive properties, such as anti-oxidation, anti-proliferation, anti-inflammation and induction of apoptosis [104]. Specifically, the anti-oxidative efficacy of resveratrol to prevent the ROS generation and oxidative stress that may drive epithelial cells into an EMT program can be an effective characteristic of resveratrol to prevent the EMT of cancer cells. Actually, modulation of oxidative stress may be an efficient therapeutic tool for the inhibition of cancer progression [105]. Resveratrol inhibited the hypoxia-enhanced proliferation, invasion and EMT process in Saos-2 osteosarcoma cells via downregulation of the HIF-1α protein [106]. A previous study also revealed that resveratrol effectively suppressed the hypoxia-driven ROS-induced invasive and migratory ability of pancreatic cancer cells by inhibiting the Hh signaling pathway, which is able to regulate the EMT [107].

In addition, a recent finding indicated that resveratrol suppressed invasion and metastasis in gastric cancer by inhibiting the Hh signaling pathway and EMT [108]. In PC-3 and LNCaP prostate cancer cell lines, lipopolysaccharide (LPS) was used to trigger EMT, but resveratrol inhibited LPS-induced morphological changes, cell motility and invasiveness, the expression of EMT markers and inhibited the expression of glioma-associated oncogene homolog 1 (Gli1), suggesting that resveratrol has in part the ability to inhibit the EMT process through the Hh signaling pathway [109].

Similar to genistein, resveratrol abrogates the TGF-β1-induced EMT process for cancer progression. In LoVo colorectal cancer cells, resveratrol inhibited the invasive and migratory ability of LoVo cells, increased the expression of *E*-cadherin and repressed the expression of vimentin, via the inhibition of the TGF-β1/Smads signaling pathway [110]. Other studies also support the role of resveratrol in EMT inhibition. Xu *et al.* reported that resveratrol reversed EMT by inhibiting AKT signaling in pancreatic cancer [111]. Previously, AKT1 was also shown to promote EMT, as well as to increase metastasis in squamous cancer and sarcoma [112]. On the contrary, AKT1, but not AKT2 and AKT3, inhibited EMT in breast cancer, depressing Twist1 activation [112]. In the MCF-7 breast cancer cell line, resveratrol was found to inhibit EGF-induced EMT via inhibition of the EGF-mediated Erk pathway activation [113]. The role of resveratrol in inhibiting EMT induction was demonstrated in A549 lung cancer cells [114].

### 3.3. Kaempferol

Kaempferol (3,5,7-trihydroxy-2-(4-hydroxyphenyl)-4*H*-1-benzopyran-4-one) is one of the flavonoids found in many edible plants, like tea, broccoli, cabbage, beans and tomato, and its name was derived due to its specific source of the rhizome of *Kaempferi galangal* L., known as a popular traditional aromatic plant [115,116]. As one of the phytoestrogens due to its polyphenolic structure, kaempferol also exerts anti-proliferative and anti-carcinogenic actions though ER, AR, the aryl hydrocarbon receptor (AhR) and the progesterone receptor (PR) signaling pathways in many types of cancer [117,118,119].

Apoptosis is one of the main pathways for kaempferol to induce the anti-carcinogenic effect. In some cells, kaempferol induced apoptosis by stimulating the enzyme activity of caspases, which are a group of the cysteine proteases that are important initiators or effectors of the apoptosis process [65]. For caspase-3, it activates apoptosis by inducing DNA fragmentation and chromatin condensation in nucleus [120]. Kaempferol decreased the mitochondria potential by the stimulation of caspase-3 activity, resulting in the apoptosis of human lung non-small carcinoma cells [121,122]. In caspase independent pathways, kaempferol also promoted apoptosis by translocating apoptosis-inducing factor (AIF) into nucleus. AIF, which exists mainly in the space between the inner and outer mitochondrial membrane, was translocated into nuclei by kaempferol to induce nuclear condensation and large-scale DNA fragmentation [123,124].

Kaempferol also seems to inhibit cancer invasion and metastasis via the inhibition of EMT. Specifically for lung cancer, kaempferol was well known to suppress cancer migration, invasiveness and metastasis by modulating the expression of EMT proteins. Kaempferol significantly reduced the expression of MMP and mesenchymal markers and repressed metastasis and EMT by the TGF-β-dependent signaling pathway in non-small cell lung cancer [125]. In A549 lung cancer cells, kaempferol exerted the suppression of TGF-β1-induced EMT, migration and metastasis by blocking Smad3 as an important mediator of TGF-β signaling. In this study, PI3K/Akt signaling stimulated EMT and cell migration by directly phosphorylating Smas3, but kaempferol repressed EMT and cell migration by inhibiting Akt1-mediated phosphorylation of Smad3 [126]. The effect of kaempferol on EMT in relation to cancer progression has not been fully demonstrated yet, except for lung cancer. Although not in cancer, kaempferol was found to alleviate fibrotic airway remodeling via bronchial EMT by modulating protease-activated receptor-1 (PAR1) activation, which was entailed by TGF-β, suggesting that it may be a potential therapeutic agent targeting asthmatic airway constriction [127]. Since kaempferol is considered to have obvious anti-EMT efficacies, it will be applicable to other cancers for the prevention of cancer metastasis induced by EMT. According to a recent review on ribosomal S6 kinase (RSK) isoforms, the synthetic version of kaempferol, kaempferol-glycoside, can effectively target the invasion and metastasis of cancer by inhibiting RKS isoforms that promote invasion and tumor metastasis [128].

### 3.4. DIM

*Brassica* vegetables, such as cabbage, Brussels sprouts and broccoli, are rich in indole-3-carbinol (I3C), which is one of the active phytonutrients and a precursor of many different compounds, especially DIM. I3C undergoes acid-catalyzed dehydration and polymerization to be DIM in an acidic environment [129].

DIM has been shown to abrogate the proliferation of human cancer cells of prostate, breast, colon, ovary and pancreas [130]. Especially, it has the potential role of AhR signaling by acting as one of the selective AhR modulators (SAhRMs) in mammary carcinogenesis prevention [131]. With respect to anti-cancer mechanisms, DIM encourages apoptotic cell death by downregulation of NF-κB, survivin and Bcl-2 as anti-apoptotic factors and upregulation of Bax, a pro-apoptotic factor [132,133]. One surprising ability of DIM as a potential anti-cancer compound is its selective induction of apoptosis in cancerous cells, but not in normal cells [134]. Furthermore, DIM activates cell cycle modulators, p21 and p27, leading to G1 cell cycle arrest in breast, ovarian, prostate and colon cancer cell lines [132,135].

In addition to the protective effects of DIM against tumorigenesis, DIM has more effects in inhibiting chemotaxis and metastasis by inactivating of CXCR4 and CXCL12 at low concentrations by affecting AhR and ER in carcinogenesis [136]. The urokinase plasminogen activator (uPA) system is confirmed to have potential effects in cell migration, angiogenesis, cancer invasion and metastasis. Some studies have shown that DIM can downregulate uPA in the inhibition of tumor progression in prostate and breast cancer [137,138]. Furthermore, DIM inhibited cell proliferation, migration and metastasis by directly inactivating vascular endothelial growth factor (VEGF) and MMP and by involving the degradation of the basement membrane in original vessels, endothelial cell activation and migration [139].

DIM has been reported to repress tumor malignancy via inhibiting the EMT process. DIM deterred EMT in prostate cancer cells by blocking AR signaling and the expression of prostate-specific antigen (PSA), an AR-target gene, resulting in the reduction of the expression of EMT markers, ZEB1, *N*-cadherin and fibronectin [140]. DIM upregulated the protein expression of *E*-cadherin and downregulated the protein expression of vimentin by attenuating miR-92a and the NF-κB receptor activator. The PI3K/Akt/mTOR/NF-κB signaling pathway has been known to induce migration, invasion and EMT. However, DIM reversed EMT by modulating the PI3K/Akt/mTOR/NF-κB signaling. DIM also decreased the expression of mesenchymal markers ZEB1, vimentin and Slug and changed the morphology of pancreatic cancer cells into the epithelial form [141,142].

Recently, miRNA has been shown to have a crucial role in the initiation, progression and metastasis of cancer, and DIM has been shown to function as miRNA regulators affecting cancer metastasis and growth by modulating EMT [143]. DIM can repair the miRNA deformity and inhibit the mutation of miRNA in vinyl carbamate-induced mouse lung cancer [144]. According to a recent study by Li *et al.*, DIM treatment decreased the expression of vimentin, ZEB1 and Slug as EMT markers with reversal of EMT in pancreatic cancer cells via the increased expression of miR-200 [145]. DIM promoted the expression of the let-7 family, while it reversed the tumor progression and EMT by preventing ZEB1 [145], and it conducted the reduction of enhancer of zeste homolog 2 (EZH2) intervening in EMT phenotypic cells [146]. In a recent study, DIM also showed depression of miR-34a and inhibition of cancer growth via AR signaling *in vivo* [147]. In addition, DIM upregulated the protein expression of *E*-cadherin and downregulated the protein expression of vimentin by attenuating miR-92a and the NF-κB receptor activator [148]. In this way, DIM as an miRNA controller can lead to the repression of EMT and to a new therapy in prostate cancer.

Li *et al.* also demonstrated that DIM could be effective against pancreatic cancer by reversing the EMT phenomenon by upregulating the expression of miR-200 and the let-7 family, which are typically lost in many other cancers [145]. Wu *et al.* showed that DIM inhibited the metastasis of nasopharyngeal carcinoma (NPC) and EMT by promoting the expression of mesenchymal markers, Snail and Slug [149]. Moreover, DIM reversed *E*-cadherin expression in NPC, which experienced the EMT process, and DIM-induced *E*-cadherin expression has confirmed as having a significant positive correlation with the long-time outcome of NPC patients [150].

## 4. Conclusions

The phytoestrogens have generated considerable interests as alternatives for hormone replacement therapy (HRT) and chemopreventive compounds for recent years, because they have diverse bioactivities, low toxicity for the human body and easy acceptance as dietary supplements.

**Figure 2:**
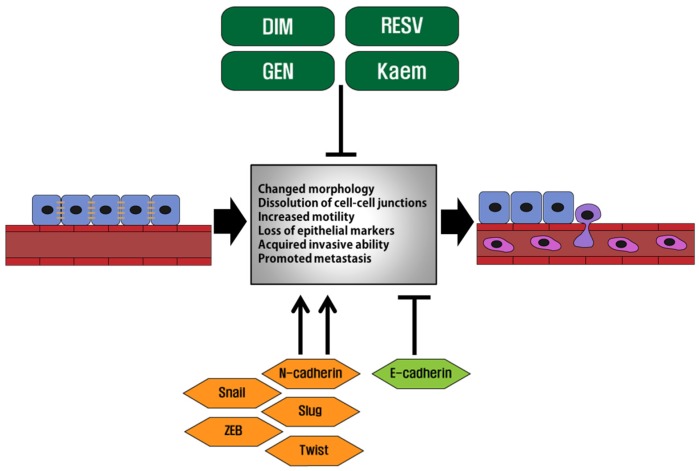
Schematic diagram of the EMT process and the roles of phytoestrogens, genistein, resveratrol, kaempferol and DIM, in the regulation of the EMT process in cancer metastasis. EMT plays a key role in tumor progression. The cells undergoing EMT show *E*-cadherin repression, but increased expression of EMT markers, such as Snail, Slug and vimentin, and cell motility-related proteins, including MMPs and uPA. As a result, they gain enhanced migration and invasion capabilities: primary cancer cells lose cell-cell adhesion, break through the basement membrane and enter the bloodstream through intravasation. Later, the circulating tumor cells exit the bloodstream to migrate to the specific metastatic sites, where they undergo MET for clonal outgrowth. On the other hand, genistein, resveratrol, kaempferol and DIM may inhibit cancer metastasis by repressing the EMT process through affecting the signaling pathways associated with EMT and regulating the expression of EMT markers.

In the present study, we suggested significant roles of phytoestrogens in the inhibition of cancer progression via EMT, which is a crucial process inducing cancer migration, invasion and metastasis. Genistein, resveratrol, kaempferol and DIM have been evaluated to effectively repress the EMT process in diverse cancers by affecting the signaling pathways and the expression of EMT-related markers, as shown in the diagram of Figure 2. Specific signaling pathways and EMT markers regulated by genistein, resveratrol, kaempferol and DIM, are summarized in Table 1. In addition, since these phytoestrogens have been also reported to exert anti-carcinogenic effects by controlling cell cycle, cell proliferation and apoptosis in hormone-related, as well as non-hormone-related cancers, they may be considered as effective anti-cancer agents to inhibit the whole process of cancer progression.

Nevertheless, controversies about the chemopreventive effect of phytoestrogens have existed because their cancer promoting effects have been also reported. Several phytoestrogens, including genistein and resveratrol, were known to have a biphasic effect, especially in hormone-dependent cancers: based on the specific concentration, cancer cell growth is stimulated at lower concentrations and inhibited at higher concentrations [19]. More skepticism regarding the chemopreventive effects of phytoestrogens came from the *in vitro* concentration of phytoestrogen being generally 10 μM, and plasma concentrations of >10 μM cannot be attainable by the dietary intake of phytoestrogen-containing food; thus, low levels of phytoestrogen, rather, may stimulate cancer growth. However, this insistence is still incompetent to abrogate the experimental and epidemiological evidence of phytoestrogens supporting anti-proliferative and anti-carcinogenic effects. Accordingly, more investigations of the relationship between the hazardous and chemopreventive effects of phytoestrogens in the body are needed at this stage. Besides, the poor bioavailability of phytoestrogens, such as resveratrol, in the body is another pitfall in the application of phytoestrogens, which has to be improved [97]. The toxicological issues posed by phytoestrogens also should be considered in their applications. For instance, the possibility that serum genistein concentrations found in soy-fed infants may induce thymic and immune abnormalities was raised [151]. High doses of genistein (>5 μM) may act as a topoisomerase II inhibitor and a DNA damaging genotoxin [152]. Genistein was reported to be capable of altering the toxicological behaviors of the endocrine-active pesticide methoxychlor and likely other endocrine-active compounds, as well [153]. In this way, the adverse effects of phytoestrogens on normal cellular function have been found. As with many other compounds, there are many pros and cons associated with phytoestrogens, and thus, it is urgently needed to shape the development of guidelines for the use of phytoestrogens for maximizing health benefits, as well as minimizing the adverse effects [154].

From this review, we may suggest that phytoestrogens are potent compounds that abrogate the cell migration, invasion and metastasis of cancer by effectively suppressing the EMT process. Phytoestrogen treatments are a promising way to prevent cancer development as safer alternatives for the natural strategies against cancers, though we need more research and interest for going forward to clinical trials.

## Figures and Tables

**Table 1:** Potential signal transductions related to EMT targeted by dietary phytoestrogens.

Phytoestrogen	EMT-Related Signalings	Reference
Genistein	TGF-β, Smad, PI3K, Akt, NF-kB, Notch-1, MAPK, ER	[58,90,92]
Resveratrol	Hedgehog, TGF-β, Smad, AKT, EGF	[107,108,110,111,113,114]
Kaempferol	ER, AR, AhR, PR, TGF-β, Smad3, PI3K/Akt, RAF/ERK	[117,118,119,125,126,128]
Diindolylmethane	AR, PI3K/Akt/mTOR/NF-κB, Hedgehog, miR-200, RANKL, β-catenin	[140,141,143,145,147,148,150]
